# Cost-effective and versatile Hardware-in-the-Loop system for DC/DC converter emulation in education and research

**DOI:** 10.1016/j.ohx.2025.e00701

**Published:** 2025-09-19

**Authors:** Alejandro Gaviria-Cano, Cristian Escudero-Quintero, Jose David López-Suárez, Juan Pablo Villegas-Ceballos, Elkin Edilberto Henao-Bravo, Sergio Ignacio Serna-Garcés

**Affiliations:** Facultad de Ingeniería, Institución Universitaria ITM, Medellín, 050041, Colombia

**Keywords:** DC/DC converter emulation, Hardware-in-the-Loop (HIL), Energy conversion, Power electronics, Educational tool

## Abstract

This work presents a cost-effective, versatile hardware-in-the-loop (HIL) system for non-isolated DC/DC converter emulation. The system emulates five types of converters (Buck, Boost, Buck-Boost, Cuk, and SEPIC) and targets educational and research applications. The hardware uses an ARM Cortex M7 microcontroller to perform real-time calculations and produce analog signals that emulate the behavior of the converters. Its economical, compact, and modular design is optimized to facilitate use in educational environments and research projects with limited resources. Additionally, the system includes software that enables automatic configuration of the code necessary for emulation, offering flexibility while reducing costs and complexity

## Graphical abstract

**Figure:**
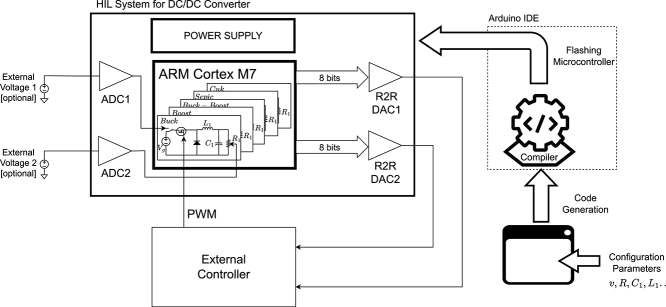

## Specifications table

**Table:** 

*Please replace the italicized instructions in the right column of the table with the relevant information about your hardware.*	

Hardware name	*HILDCxLab: Hardware-in-the-Loop DC Converter Experimental Laboratory*

Subject area	Educational tools and open source alternatives to existing infrastructure

Hardware type	Electrical engineering and computer science

Closest commercial analog	OPAL-RT Technologies Typhoon HIL dSPACE PLECS RT Box

Open source license	CERN Open Source License Permissive (CERN-OHL-P)

Cost of hardware	$275

Source file repository	http://doi.org/10.17605/OSF.IO/WJV75

## 1 Hardware in context

It is important to teach DC/DC converters topics in power electronic courses for electrical and electronic engineering bachelor programs due to their wide application in power systems, electronic devices, and renewable-based generation sources. These converters adjust voltage and current levels to supply devices, optimize power processing, and ensure compatibility between different sources and loads [1]. However, in an educative environment, students may face several challenges when assembling these systems, such as complexity in circuit design, selection of suitable components (e.g., inductors, capacitors, and transistors), fabrication and assembly times, difficulties in understanding the dynamic behavior of the converter and implementation of control strategies [2], [3]. Because of this, one strategy for teaching and adjusting preliminary designs is implementing Hardware-In-the-Loop (HIL) strategies in the classroom, whereby the student can perform the converter and control designs. Once the designs are adjusted, it is possible to perform the experimental setup [4], [5], [6].

Commercial simulation systems homologous to the proposed hardware, such as OPAL-RT [7], Typhoon HIL [8], dSPACE [9], and PLECS RT Box [10], are advanced tools widely used in power electronics system research and development, which offer high accuracy and real-time simulation capabilities, making them ideal for industrial applications and high-demand projects in advanced research environments. However, their cost is significantly high, making them less accessible for educational environments or those with limited budgets. In addition, these platforms often require a considerable amount of time to learn their proper management due to the complexity of their configuration and operation, requiring additional training for users [11].

This paper presents a hardware design that allows the emulation of five common DC/DC converter types: Buck, Boost, Buck-Boost, Cuk, and SEPIC. This device, developed specifically for educational and research applications, offers a practical and economical solution that facilitates access to experimentation in power conversion. It provides a fundamental resource in both teaching and research, as it allows students and professionals to experiment with different configurations without the risks and costs associated with high-power hardware [12], [13].

In contrast, the proposed hardware offers an affordable and economical alternative for DC/DC converter emulation in educational and academic research applications. Unlike commercial systems, where designers create for versatile use in several industrial applications, we explicitly optimized our design to emulate five converter topologies (Buck, Boost, Buck-Boost, Cuk, and SEPIC) [14], thereby reducing complexity and cost. The developed hardware is based on a microcontroller that receives external PWM signals and, through real-time calculations, provides discrete solutions to the voltage and current equations associated with the selected converter. These solutions are sent through digital-to-analog converters (DACs), providing real-time analog outputs that emulate the converter’s behavior. In this way, the device allows users to evaluate variations in design parameters and analyze the system’s dynamic response to changing operating conditions without building each converter physically. In addition, the hardware includes software that quickly configures the system and enables the automatic generation of the emulation code for the selected converter. This approach gives the user great flexibility, allowing them to test different converter topologies and classical control strategies without costly hardware modifications.

## 2 Hardware description

**Fig. 1:**
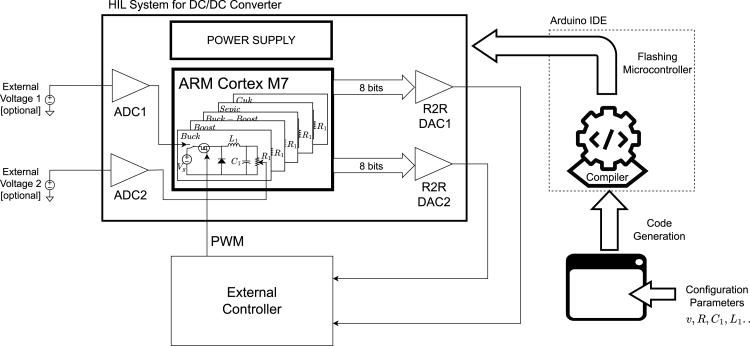
Emulation hardware block diagram for DC/DC converters.

This work presents a dedicated hardware platform designed to enable precise and efficient emulation of switched-mode DC/DC converters, focusing on educational and research applications in power electronics and renewable energy. Unlike conventional emulation systems, which are typically robust yet complex and less accessible, the proposed device features a compact and streamlined architecture. The design emphasizes ease of integration and user-friendliness while maintaining the accuracy and responsiveness essential for real-time converter emulation. As a result, it significantly reduces the complexity associated with hardware-in-the-loop (HIL) implementations, lowering the entry barrier for both academic and experimental use. The simplified schematic of the proposed hardware is presented in the Fig. 1 and key features and advantages are summarized as follows:

• Users can quickly set up the system without specialized software. The interface simplifies the emulation process, reducing setup time and complexity.
• The hardware precisely reproduces the fast-switching behavior of DC/DC converters, which is critical for analyzing systems with rapid transitions.
• With dimensions of 15 cm × 10 cm and the ability to operate directly from the AC grid, the device is ideal for laboratory environments. Its architecture minimizes the risk of short circuits and reduces electrical noise, enhancing user safety and preventing common setup errors.
• A programmable core emulates several power devices, including solar panels and wind turbines. This facilitates scalable laboratory setups and comprehensive renewable energy scenarios.
• The proposed hardware offers a lower-cost and simpler alternative to existing emulation platforms, enabling students and researchers to focus on system analysis and design.
• The graphical interface supports the configuration of up to five preloaded converter topologies. Users can define a constant input voltage level or change its value, adjust energy storage elements (inductance, capacitance, load resistance). Once configured, the system automatically generates the converter’s programming code.
• Two digital inputs allow PWM-based converter switching control. State variables are computed in real-time and reproduced using resistor-based DACs, enabling feedback to external controllers for advanced control strategies.
• The system operates via USB or directly from the AC grid, offering laboratory and standalone scenarios flexibility.

## 3 Hardware/software requirements modeling

**Fig. 2:**
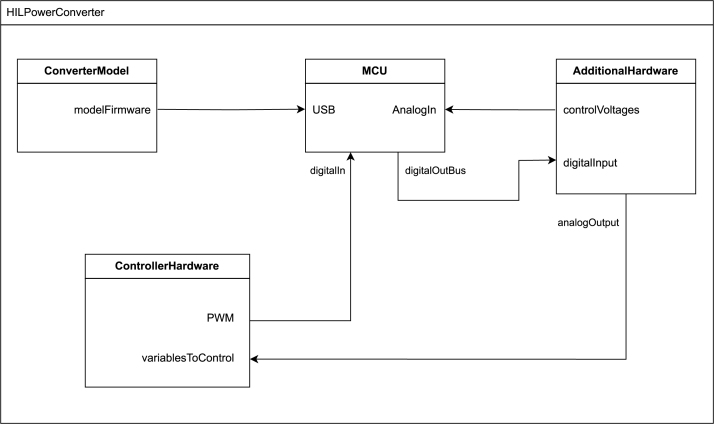
Physical model based on components of the HIL simulator of DC/DC switching converters.

The physical model represents the concrete implementation of the system, detailing its physical components, their properties, and how they interact to meet the functional requirements. With this model, it is possible to visualize how the logical designs will materialize, ensuring that the physical components (DACs, processors, etc.) comply with the specifications. Specifically, the HIL emulator was divided into four components (see Fig. 2): the converter model generator (ConverterModel), the converter emulator (MCU), the emulated converter controller (ControllerHardware), and additional hardware containing the DACs and the input voltage and load variation (AdditionalHardware).

### 3.1 Description of requirements

The requirements to be implemented by each component are shown in Table 1. The ConverterModel component is in charge of generating the model of the converter to be emulated using a graphical interface (GUI – Graphic User Interface) that allows the user to select one of five converters and the value of its parameters: inductor, capacitor, load, and switching frequency. The component must generate the C code of the asynchronous model of the selected converter, which must comply with the language standard defined in ISO/IEC 9899: 2018. This component is a software module developed in Python. The generated converter model is deployed in the Arduino integrated development environment (IDE). It is compiled to obtain a file with a HEX extension to download on the microcontroller that will emulate the converter.

The converter emulator shall be a Microcontroller Unit (MCU) that satisfies requirement #3. Most MCU constraints, such as architecture, CPU, FPU, instructions per cycle, and speed, are related to performance and accuracy. Due to the inherent dynamics of DC-DC converters, i.e., they have switched circuits at frequencies on the order of tens of kHz, swift instruction execution is required, ideally with parallel processing and high-accuracy computational units that minimize quantization error.

**Table 1:** Description of system requirements.

**Description**	**Generate in C language models of second and fourth order DC/DC switching converters using the forward Euler method.**

ID	1

Type	Functional

Constraints	**Converters to simulate:** buck, boost, buck-boost, SEPIC and Cuk.
	**Model:** switched no losses.
	**Parameters:** L, C, R, Fsw.
	**Generate C code according to ISO/IEC 9899:** 2018 standard.

Implemented by	ConverterModel

**Description**	**Model generator application with GUI.**

ID	2

Type	Non-Functional

Constraints	App built in Matlab, Python or Java Script.

Implemented by	ConverterModel

**Description**	**Simulate the dynamic behavior of second and fourth order DC/DC switching converters. Real-time, high-precision numerical simulator.**

ID	3

Type	Functional and non-functional

Constraints	**Architecture:** ARM.
	**CPU:** 32 bits.
	**FPU:** 32/64 bit.
	**Instructions per clock cycle:** 2@25%
	**Speed:** 600 MHz ∼ 1.008 GHz (Overclock, cooling requer’d).
	**Flash memory:** 4 MB.
	**RAM memory:** 1 MB.
	**ADC resolution:** 10 bit.
	**Sampling frequency:** 1 MSPS.
	**ADC channels:** 2.
	**ADC Vref:** 0–3.3 V.
	**USB version:** 2.0
	**I/O pin:** 18
	**Analog input:** 8

Implemented by	MCU

**Description**	**Provide two simulated switching converter state variables for low-cost, real-time processing.**

ID	4

Type	Functional and non-functional

Constraints	**DAC resolution:** 8 bits.
	**DAC type:** R-2R ladder.
	**DAC channels:** 2
	**DAC Vref:** 3.3 V.

Implemented by	AdditionalHardware

**Description**	**The system has the ability to adjust the input voltage and converter load.**

ID	5

Type	Functional

Constraints	**Output:** Analog.
	**Channels:** 2.
	**Output voltage range:** 0.0–3.3 V.

Implemented by	AdditionalHardware

**Description**	**High-performance, low-cost asynchronous or synchronous DC/DC switching converter controller.**

ID	6

Type	Functional and non-functional

Constraints	**Type:** Assymetric PWM.
	**Frequency:** Greater than 50 kHz.
	**Duty cycle:** [10%, 90%].
	**ADC sampling frequency:** Greater than 1 MSPS.
	**ADC channels:** 2.
	**CPU:** 32 bits.
	**FPU:** 32 bits.

Implemented by	ControllerHardware

In the context of real-time emulation of power converters using microcontrollers, both software execution speed and hardware limitations play a critical role. The emulated system in this work is a lossless asynchronous Buck converter, modeled using discrete-time integration methods and executed on an ARM Cortex-M7 microcontroller (Teensy 4.1).

A typical iteration of the model involves several floating-point multiplications and additions, digital input reading of the PWM signal, and bit-wise manipulation to generate analog-equivalent outputs through a resistive DAC. Based on the microcontroller’s instruction timing, a single Euler step for a second-order system like the Buck converter requires approximately 7 floating-point multiplications, 4 additions, 1 digital read, and two 8-bit GPIO writes. Considering the hardware floating-point unit (FPU), memory access latency, and GPIO operations, each iteration takes approximately 250 clock cycles.

In hardware-in-the-loop (HIL) systems, a commonly accepted rule to preserve waveform fidelity and prevent aliasing-induced sub-harmonic oscillations is to simulate the plant model with at least 10 integration steps per switching period [15]. For a switching frequency of 100 kHz (i.e., a 10μs switching period), this results in 1μs available per integration step. To execute 250 clock cycles within 1μs, a minimum processor speed of 250 MHz is required.

The Teensy 4.1, based on the ARM Cortex-M7 core, supports clock frequencies up to 1 GHz, although active cooling is required at that level. Operating the microcontroller at 600 MHz offers a practical compromise, providing sufficient computational headroom to simulate converters at high switching frequencies with oversampling, handle GPIO operations reliably, and support real-time controller execution or communication without compromising performance. This also mitigates the appearance of aliasing artifacts such as low-frequency sub-harmonic oscillations, which are known to arise when the simulation step size approaches the switching period of the converter [15], [16].

The emulator will only calculate two state variables, no matter if the converter is a fourth-order converter. Therefore, it requires two analog output channels (DAC - Digital to Analog Converter). The analog outputs of the HIL emulator are generated using 8-bit R-2R ladder DACs, chosen for their simplicity, low cost, and high-speed performance. This resolution provides 256 discrete levels, which we determined sufficient to reproduce the average and dynamic behavior of the key state variables (e.g., capacitor voltage and inductor current) for educational and experimental validation purposes.

The system’s design balances functional performance, cost, and implementation simplicity. To emulate the behavior of two state variables in real-time while maintaining low cost, an 8-bit parallel DAC was selected. This resolution meets the non-functional requirements of fast digital-to-analog conversion and affordability, making the hardware accessible and easy to reproduce.

While the 8-bit resolution is sufficient for most educational and experimental applications, higher signal fidelity may be desirable in scenarios requiring finer detail or more accurate ripple reproduction. In such cases, users can readily upgrade the system due to its open hardware architecture. Specifically, they may replace the existing DACs with 10-bit or serial DACs, thereby improving resolution without significantly increasing design complexity. Furthermore, future versions may natively integrate higher-resolution DACs to enhance precision while maintaining bandwidth and cost efficiency. Similarly, selecting a 10-bit ADC reflects a compromise between accuracy and cost. Although not exceptionally high, this resolution is adequate for adjusting the input voltage and load conditions of the emulated DC/DC converter with sufficient precision. These values are acquired through the ADC channels and contribute to the emulation’s dynamic response.

The USB interface plays a central role in system flexibility. It receives the C program generated by the ConverterModel component, which defines the converter’s operational parameters. The emulator’s performance relies on memory availability, instruction parallelism, and processor architecture to meet functional and non-functional requirements, such as high numerical precision and real-time execution.

The controller requirements were defined based on Arduino Microcontroller commonly used for converter control, as they are not part of the emulation hardware. They are restricted to Microcontroller with at least two PWM outputs at frequencies of 100 kHz and two ADC channels with a sampling rate 10 times the PWM signal frequency and 8-bit resolution.

The proposed HIL system involves three distinct time bases that are critical for proper emulation performance:

**Fig. 3:**
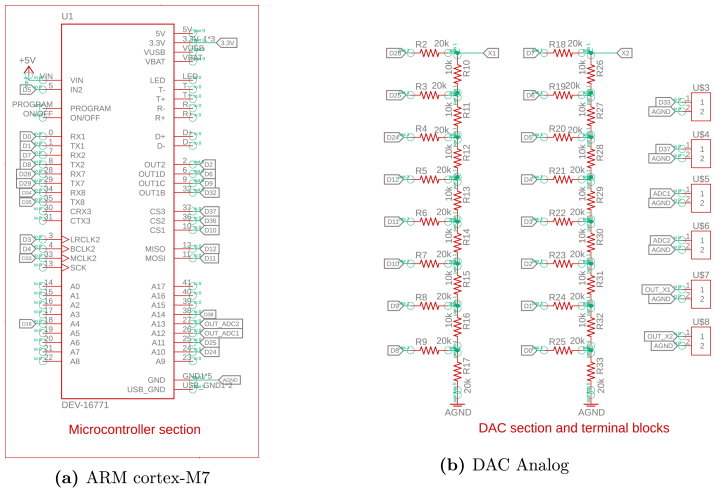
Schematic Part A.

• Input Sampling Time (PWM Inputs): The hardware receives external PWM control signals from a DSP or microcontroller, typically operating at switching frequencies of up to 100 kHz. In the present implementation, the PWM inputs are not sampled only once per model step; instead, multiple instantaneous readings are taken in the interval between consecutive executions of the HIL model, with a temporal resolution of approximately 10 ns. These samples are processed using the Input Oversampling Method (IOM) proposed by Zamiri et al. [15], [16], which integrates and corrects duty-cycle variations within that period, providing a representative binary input for the next simulation step. This approach improves duty-cycle reconstruction compared to single-sample acquisition, although all sampling and processing occur sequentially with respect to the model execution.
• HIL Model Sampling Time: The converter models are discretized using the forward Euler method and executed in real time on a Teensy 4.1 microcontroller, which incorporates a 600 MHz ARM Cortex-M7 processor. For each model, the total number of clock cycles per iteration determines the achievable update rate (e.g., approximately 15 cycles for second-order models and 26 cycles for fourth-order models). In the current configuration, the model step is set to 1μs, meaning the system executes the equations, applies the processed IOM duty-cycle value, updates the outputs, and then repeats the sampling–processing–execution loop.
• Output Update Time (DAC Refresh Rate): The inductor current and capacitor voltage are output through two 8-bit R–2R ladder DACs. These signals are updated synchronously at the end of each model step (currently every 1μs) immediately after computing the new state variables from the discretized equations. The DAC update rate therefore matches the HIL model sampling time, ensuring consistent timing between computation and output refresh, but also inheriting any timing limitations from the sequential processing structure.

Given this synchronization of input sampling, model computation, and output refresh, the system ensures real-time performance up to a maximum recommended switching frequency of 100 kHz. Operating at higher frequencies may result in timing violations or degraded model accuracy.

## Design files

**Fig. 4:**
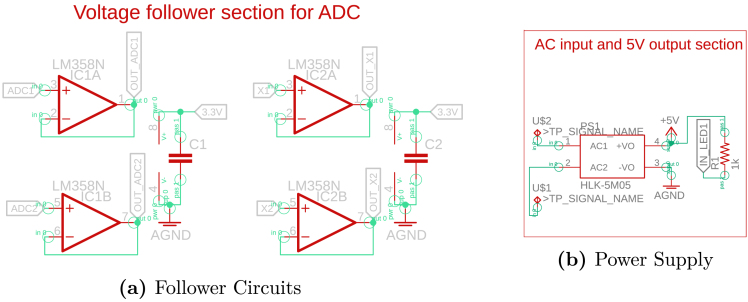
Schematic Part B.

The proposed system for power converter emulation comprises several key components, with schematics presented in Fig. 3, Fig. 4. The hardware design is intended to be modular, allowing for easy assembly, with components strategically arranged to facilitate connectivity and reduce electrical noise. The system’s core is a Teensy 4.1 microcontroller featuring an ARM Cortex-M7 processor operating at 600 MHz. This microcontroller provides multiple input/output (I/O) pins and analog inputs, making it a versatile platform. The discretized model of the power converters runs directly on Teensy 4.1, enabling precise calculations of the capacitor voltage and inductor current waveforms at high speed. The system design incorporates two resistive digital-to-analog converters (DACs) to reproduce the waveforms calculated in the microcontroller, which ensure the generation of signals with excellent accuracy and speed, facilitating the accurate emulation of the converter variables, as detailed in Fig. 3(b).

The design also includes two digital inputs that enable the connection of PWM signals. These signals can control the switching of the emulated converter within the device, further extending its functionality and versatility. The hardware also integrates a set of operational amplifiers configured as voltage followers, as shown in Fig. 4(a). These followers, built with the LM358 operational amplifier, provide high impedance between input and output signals, protecting the circuit from voltages exceeding the maximum allowed. Furthermore, they connect to both the microcontroller’s analog inputs and the DAC outputs, ensuring system stability and safety.

The system employs a Hi-Link HLK-5M05 power module to simplify power delivery, which converts AC grid voltage into a 5 V DC output. This module, shown in Fig. 4(b), provides an efficient and compact power supply suitable for the hardware’s requirements.

The design includes interactive elements such as LED indicators and push buttons, which can be configured for several applications. Additionally, terminal blocks have been integrated to facilitate access to the waveforms generated during converter emulation, allowing practical and flexible measurements based on the configurations selected in the microcontroller software.

**Fig. 5:**
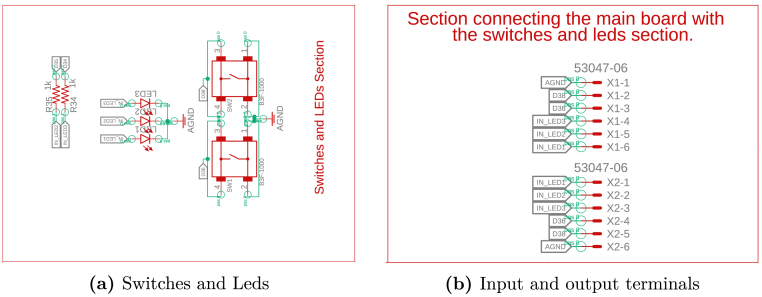
Schematic Part C.

## 4 Design files summary

The design files for this hardware are available so that they can be replicated and customized. The table below shows the file name, type and location of the design files (see Table 2) :

**Table 2:** HILDCxLab design files.

Design filename	File type	Open source license	Location of the file
BOOM_ConverterEmulator	*BOOM*	*CERN-OHL-P*	*Hardware/BOOM*
ConverterEmulator (.f3d) (.fbrd) (.fsch)	Fusion360, PCB design Figs. 3, 4, 5	CERN-OHL-P	Hardware/Shematic and layout
ConverterEmulator (brd.pdf) (sch.pdf)	PDF PCB design Figs. 3, 4, 5	CERN-OHL-P	Hardware/PDF
Converter_box (f3z)	Fusion360, 3D PCB design Fig. 7(a)	CERN-OHL-P	Hardware/3D
Converter_Box_Bottom (.stl)	Fusion360, 3D shell design Fig. 7(a)	CERN-OHL-P	Hardware/3D
Converter_Box_Top (.stl)	Fusion360, 3D shell design Fig. 7(a)	CERN-OHL-P	Hardware/3D
Interface (.py)	Python code of the interface Fig. 10	MIT License	Software
ArduinoCodeGeneration (.py)	Python code for arduino code generation	MIT License	Software

## 5 Bill of materials summary

The bill of materials for the printed circuit boards implemented in HILDCxLab can be found in the repository in the *“BOOM_ConverterEmulator”* document in the *Hardware/BOOM* location (see Table 3.)

**Table 3:** Bill of materials of HILDCxLab.

Qty	Value	Device	Description
2	10n	C-EU050-030X075	CAPACITOR, European symbol

3		LED5MM	LED

14	10k	R-US_R0805	RESISTOR, American symbol

3	1k	R-US_0207/10	RESISTOR, American symbol

18	20k	R-US_R0805	RESISTOR, American symbol

6	2828XX-2282834-2	2828XX-2282834-2	2 Position wire to board terminal block horizontal with board

2	53 047-06	53 047-06	CONNECTOR

2	B3F-1000	B3F-1000	Tactile switch SPST-NO top actuated through hole

1	DEV-16771	DEV-16771	RT1062 Teensy 4.1 i.MX ARM Cortex-M7 MPU embedded evaluation board – Check availability

1	HLK-5M05	HLK-5M05	AC/DC 220 V to 5 V, 5 W isolated switching step-down power supply module converter

2	LM358N	LM358N	OP AMP also LM158

2	PAD_4MM	PAD_4MM	

## 6 Build instructions

The first step in constructing the hardware involves fabricating the printed circuit board (PCB) shown in Figs. 3, Fig. 4, Fig. 5. This process requires using the available Gerber files, which include the schematic (.sch) and the board layout (.brd). These files are compatible with several PCB design and manufacturing tools, which allow users to import, edit, or update them according to specific needs or design improvements. Verify the PCB design in a simulation or preview software before fabrication to ensure all connections and components align correctly.

Once the PCB design ends, it is necessary to send the Gerber files to a PCB manufacturer or use in-house equipment. Attention should be given to selecting appropriate materials, such as the substrate thickness and copper weight, to meet the specific application’s requirements. After fabrication, inspect the PCB for defects, such as incomplete traces or misaligned vias, to ensure it meets the required specifications before assembly.

The housing must be 3D printed using the files from the repository in the Hardware/3D folder. The 3D printing file allows the housing to be customized to adapt to different configurations or experimental requirements according to the users’ needs.

Test fitting the printed circuit board into the housing to verify proper alignment and adjust the printed parts if necessary. Once everything is confirmed to fit correctly, proceed to hardware assembly following the steps below:

**Step 1. Soldering**: The soldering process begins with the most minor components to ensure precise placement and avoid obstructions caused by more significant components. Start by soldering the DAC resistors on the bottom layer of the PCB, ensuring that the orientation and values match the schematic. Use a fine-tipped soldering iron to ensure each connection is clean and well-bonded. After completing the DAC resistors, proceed to solder the LED resistors, capacitors, and operational amplifiers, verifying their placement according to the component layout. Once these smaller components are soldered, continue with the terminal blocks, connectors for LEDs and contactors, female headers for the microcontroller, and the HLK5M05 converter. Inspect the solder joints for any cold soldering or excess flux, and clean the PCB with an appropriate solvent if necessary. This step ensures a stable and reliable electrical connection throughout the circuit.

**Fig. 6:**
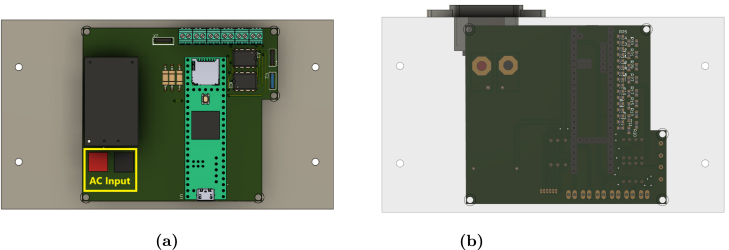
3D Model of the power converter emulator hardware (a) Top view (b) Bottom view.

**Step 2. Base assembly**: Once the PCB is fully soldered, secure the “ConverterEmulator” circuit to the lower casing (“Converter_Box_Bottom”) using screws. Align the PCB carefully with the predrilled holes in the casing to ensure a snug fit. Next, the appropriate cables are soldered to the AC input of the “ConverterEmulator”, following the wiring diagram for accuracy. Ensure that the cables are properly insulated to avoid any short circuits. Refer to Fig. 6 for guidance on this step.

**Fig. 7:**
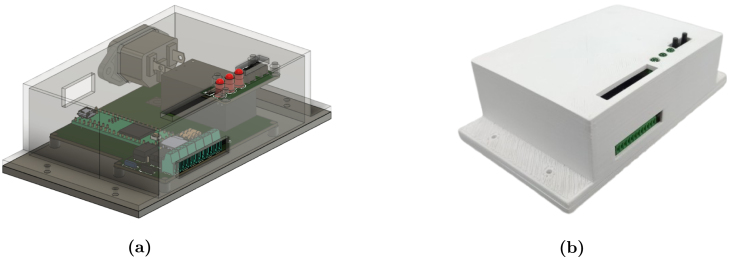
Power converter emulator (a) 3D Model of the power converter emulator hardware, (b) Final hardware assembly of the power converter emulator.

**Step 3. Final Assembly:** With the base components in place, install the AC connector into the upper casing (“Converter_Box_ Top”). Solder the connector’s terminals to the AC input on the PCB, ensuring that the polarity and connections match the schematic. After verifying the connections, test the fit by attaching the upper casing to the lower casing to ensure all components align and there is no obstruction. Refer to Fig. 7, Fig. 7 for a visual representation of the fully assembled hardware.

## 7 Operation instructions

**Fig. 8:**
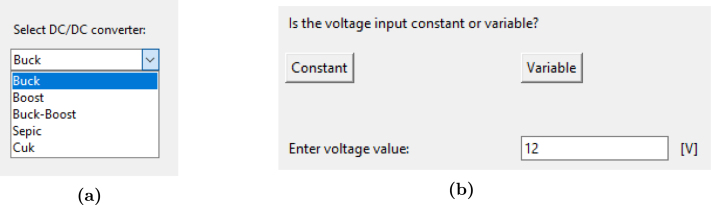
Configuration interface (a) Converter selection option, (b) Voltage type selection option.

The graphical interface allows the generation of code necessary to implement different types of converters, such as Buck, Boost, Buck-Boost, SEPIC, or Cuk. To begin, the user must select the desired converter type and click the ‘send’ button as shown in Fig. 8(a).

Once the converter is selected, the program displays a diagram of the corresponding circuit on the right side. It also enables a parameter to define whether the input voltage will be constant or variable (refer to Fig. 8(b) for this configuration option.). If a constant voltage is selected, the user can input its value; otherwise, this option will be disabled.

**Fig. 9:**
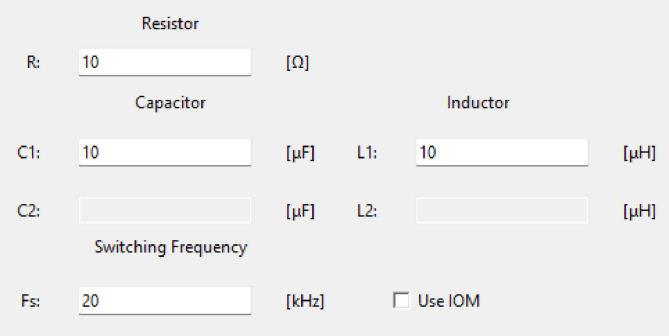
Configuration options for capacitor, inductor, voltage and load values.

In addition, the program allows the user to enter the values of the converter components in the text boxes shown in the Fig. 9:

• Resistor, expressed in ohms.
• Capacitors, with values in microfarads.
• Inductors, with values entered in microhenries.
• Switching Frequency, with values entered in kilohertz.

It is important to note that for Buck, Boost, and Buck-Boost converters, the values of components C2 and L2 cannot be modified, as these circuits only include one inductor and one capacitor.

**Fig. 10:**
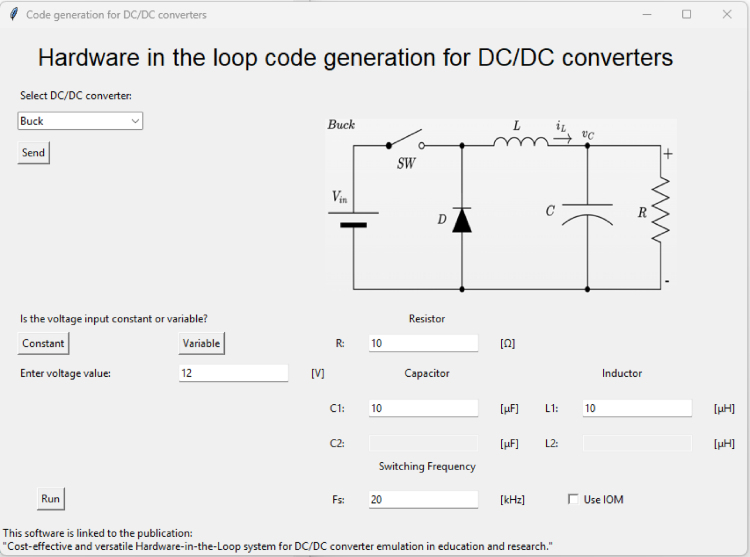
Complete configuration interface.

After configuring all the parameters, the ‘run’ button must be pressed to generate the code compatible with the Teensy 4.1. This code is saved in a folder named ‘sketch’, which must already exist in the program; otherwise, the code will not be generated correctly. Finally, the interface not only generates the code but also displays the equations associated with average values for the capacitors voltage and inductors current of the converter on steady-state operation, providing additional reference for the user. Fig. 10 shows the complete interface with all configuration fields, including an example for the Buck converter.

The analog output signals corresponding to the emulated state variables (typically capacitor voltage and inductor current) are generated through two 8-bit R-2R DACs with an output range of 0 to 3.3 V. The scaling factors applied to these signals are automatically computed by the software based on the selected converter type and the parameter values entered by the user.

For each emulation, the software analyzes the expected maximum values of the state variables and determines an appropriate linear scaling to map the internal simulation results into the DAC voltage range, ensuring optimal use of the available resolution while preventing output saturation.

Currently, the system does not allow the user to manually configure the scale or offset of the analog outputs through the interface. This design decision prioritizes ease of use and reduces complexity for students and non-expert users.

The Buck converter is modeled using a lossless, asynchronous averaged model. The state variables are the inductor current $x_{1}\left(t\right)$ and the output capacitor voltage $x_{2}\left(t\right)$. The control input is the duty cycle d(t) applied to the switch. The continuous-time state-space model is given by:

(1) $$\frac{dx_{1}}{dt}=a_{11}x_{1}\left(t\right)+a_{12}x_{2}\left(t\right)$$

(2) $$\frac{dx_{2}}{dt}=a_{21}x_{1}\left(t\right)+b_{21}u\left(t\right)$$

where:

• $x_{1}\left(t\right)$ is the output voltage,
• $x_{2}\left(t\right)$ is the inductor current,
• $u\left(t\right)=d\left(t\right)\cdot V_{in}$ is the PWM-modulated input voltage,
• $a_{ij},b_{ij}$ are coefficients derived from the circuit parameters.

To implement this model on a microcontroller for real-time emulation, the system is discretized using the forward Euler method, which approximates the derivatives as:

(3) $$x_{1}\left[k+1\right]=x_{1}\left[k\right]+T_{s}\left(a_{11}x_{1}\left[k\right]+a_{12}x_{2}\left[k\right]\right)$$

(4) $$x_{2}\left[k+1\right]=x_{2}\left[k\right]+T_{s}\left(a_{21}x_{1}\left[k\right]+b_{21}u\left[k\right]\right)$$

where $T_{s}$ is the simulation time step used in the HIL execution loop.

This discretized model corresponds directly to the implementation in the microcontroller, where each iteration updates the states $x_{1}$ and $x_{2}$ according to Eq. (4) based on the current duty cycle d[k] measured at runtime.

## 8 Validation and characterization

**Fig. 11:**
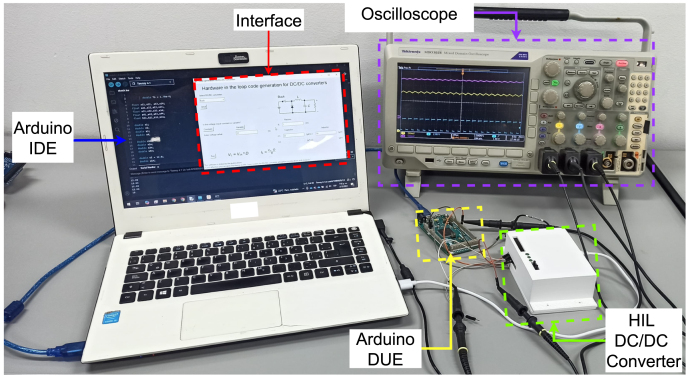
Experimental set-up for hardware performance validation.

To validate the functionality of the hardware, two of the five available converters were emulated: the Buck converter and the SEPIC converter. As a reference, prior simulations of both converters were performed using PSIM software with a switching frequency of 20 kHz. For experimental validation, the PWM signal was generated by an Arduino DUE, and the necessary simulation data were collected using a Tektronix MDO3024 oscilloscope. A comparison between the simulated data and the emulation results was conducted, ensuring the accuracy and reliability of the hardware by evaluating its performance against the simulation data. The experimental setup used to validate the hardware emulation functionality is shown in Fig. 11.

**Fig. 12:**
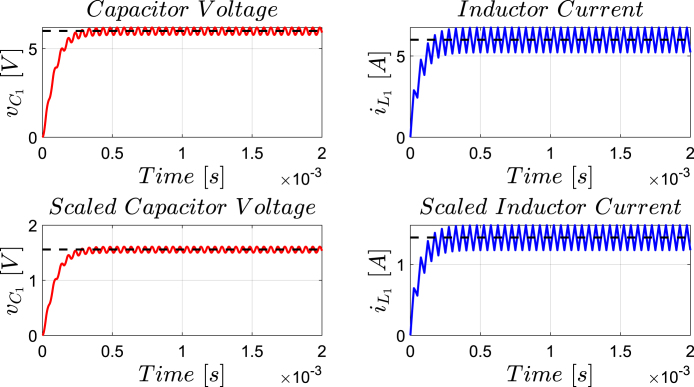
Buck converter simulation using PSIM with a 50% duty cycle for a switching frequency of 20 kHz. *Upper plot: results from the PSIM simulation. Lower plot: scaled simulation waveforms representing the target response anticipated from the HIL implementation*.

**Fig. 13:**
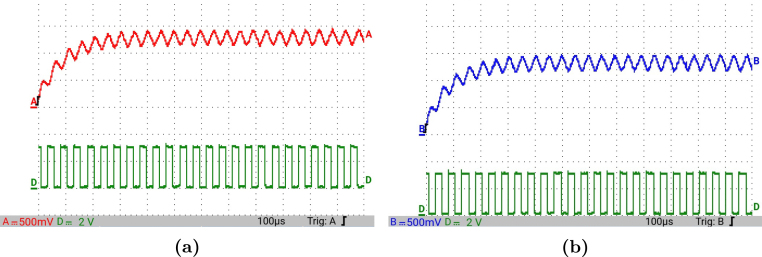
HIL testing of the Buck converter using a 50% duty cycle for a switching frequency of 20 kHz. (a) Capacitor voltage, (b) Inductor current. (For interpretation of the references to color in this figure legend, the reader is referred to the web version of this article.)

**Fig. 14:**
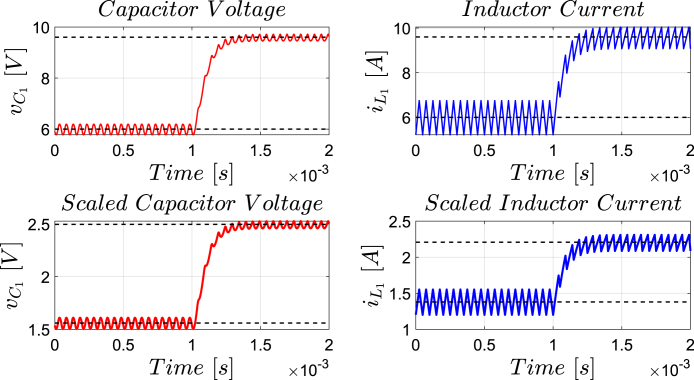
Simulation using PSIM of the Buck converter applying a change in the duty cycle from 50% to 80% for a switching frequency of 20 kHz. *Upper plot: results from the PSIM simulation. Lower plot: scaled simulation waveforms representing the target response anticipated from the HIL implementation* . (For interpretation of the references to color in this figure legend, the reader is referred to the web version of this article.)

**Fig. 15:**
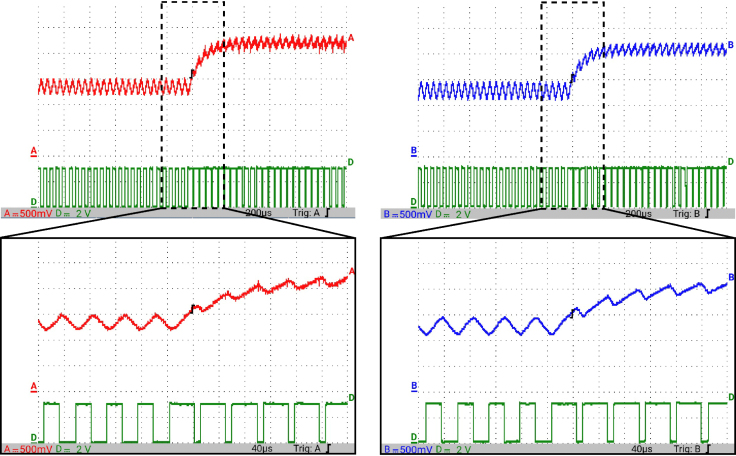
HIL testing of the Buck converter by applying a change in the duty cycle from 50% to 80% for a switching frequency of 20 kHz. Capacitor voltage (red waveform), Inductor current (blue waveform), PWM (green waveform). (For interpretation of the references to color in this figure legend, the reader is referred to the web version of this article.)

**Fig. 16:**
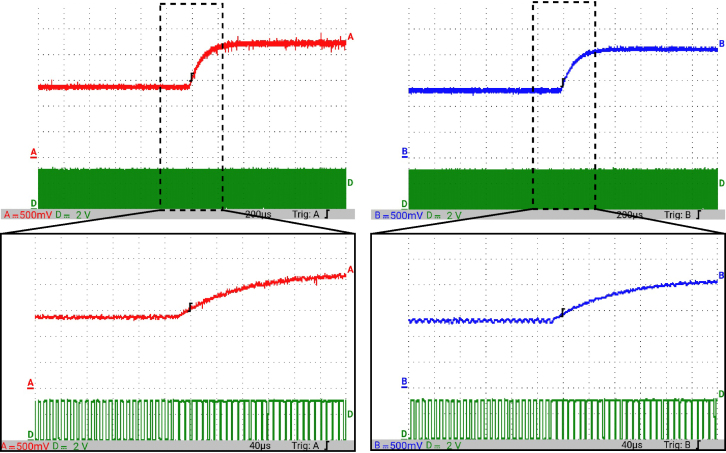
HIL testing of the Buck converter by applying a change in the duty cycle from 50% to 80% for a switching frequency of 100 kHz. Capacitor voltage (red waveform), Inductor current (blue waveform), PWM (green waveform). (For interpretation of the references to color in this figure legend, the reader is referred to the web version of this article.)

**Fig. 17:**
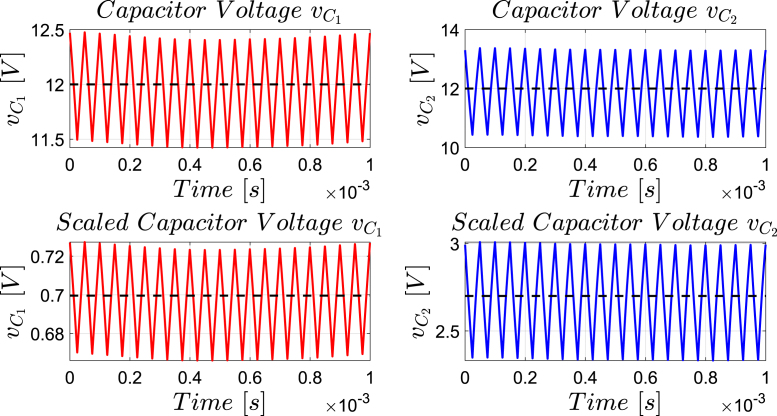
SEPIC converter simulation using PSIM with a 50% duty cycle for a switching frequency of 20 kHz. *Upper plot: results from the PSIM simulation. Lower plot: scaled simulation waveforms representing the target response anticipated from the HIL implementation*. (For interpretation of the references to color in this figure legend, the reader is referred to the web version of this article.)

**Fig. 18:**
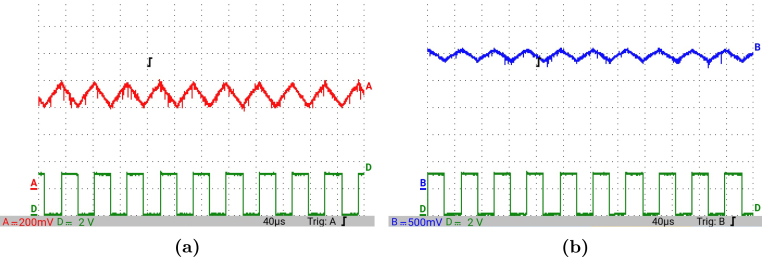
HIL testing of the SEPIC converter using a 50% duty cycle for a switching frequency of 20 kHz. (a) Capacitor voltage $C_{1}$, (b) Capacitor voltage $C_{2}$. (For interpretation of the references to color in this figure legend, the reader is referred to the web version of this article.)

**Fig. 19:**
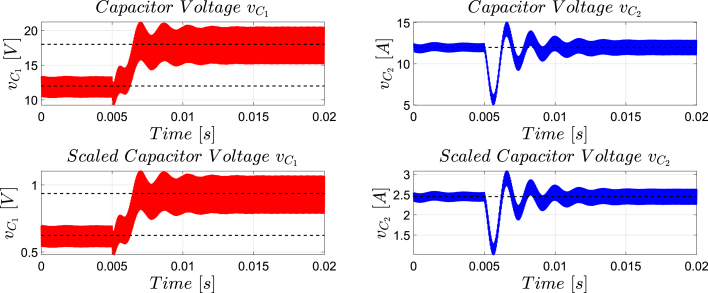
Simulation using PSIM of the SEPIC converter applying a change in the duty cycle from 50% to 60% for a switching frequency of 20 kHz. *Upper plot: results from the PSIM simulation. Lower plot: scaled simulation waveforms representing the target response anticipated from the HIL implementation*.

**Fig. 20:**
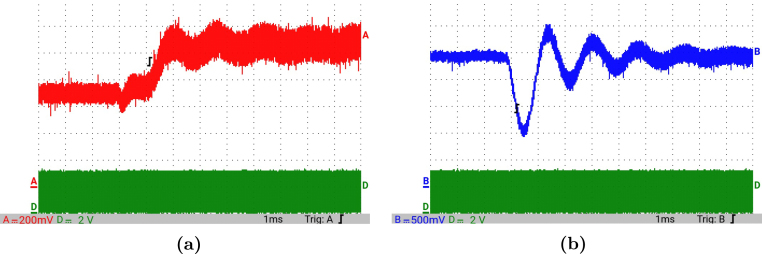
HIL testing of the SEPIC converter applying a change in the duty cycle from 50% to 60% for a switching frequency of 20 kHz. (a) Capacitor voltage $C_{1}$, (b) Capacitor voltage $C_{2}$.

**Fig. 21:**
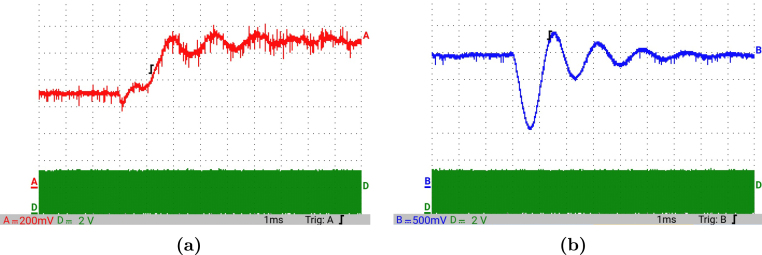
HIL testing of the SEPIC converter applying a change in the duty cycle from 50% to 60% for a switching frequency of 100 kHz. (a) Capacitor voltage $C_{1}$, (b) Capacitor voltage $C_{2}$.

• **HIL Buck converter:**
  Fig. 12 presents the simulation results obtained using PSIM for a Buck converter with the following parameters: an input voltage of 12 V, an inductor of 10 μH, a capacitor of 22 μF, a duty cycle of 50%, and a simulation time of 2 ms. In the upper graphs of the simulated results, it can be observed that the capacitor voltage ($v_{C}$) and the inductor current ($i_{L}$) reach their steady state with ripple characteristic of continuous conduction mode operation. Similarly, the lower signals in the simulation represent the same variables scaled to fit the maximum output range of the R2R DAC (3 V), corresponding to the waveforms processed by the microcontroller. For the Buck converter operating at a 50% duty cycle, the simulation results show a steady-state average inductor current of approximately 6.0 A and a capacitor voltage of around 6.0 V. The ripple in the inductor current in the simulation is approximately ±0.2 A, while the ripple in the capacitor voltage is about ±0.15 V.
  Fig. 13 presents the signals obtained from the emulated converter, including the capacitor voltage (13(a) red color), the inductor current (13(b) blue color), and the 20 kHz PWM signal generated by the external microcontroller (green color). The corresponding emulated signals yield the following scaled values: 1.38 V for the inductor current and 1.7 V for the capacitor voltage. The emulated signals exhibit comparable ripple characteristics to the simulation, although slightly attenuated due to the 8-bit DAC resolution and analog signal filtering. The measured ripple in the emulated inductor current is ±360 mV, and the ripple in the capacitor voltage is ±0.2 V. These values confirm the high fidelity of the emulation with respect to both steady-state and dynamic performance, validating the accuracy of the discretized model and the analog output system.
  To verify that the hardware is capable of emulating the dynamic behavior of the converter, a change in the duty cycle from 50% to 80% was performed. Fig. 14 presents the results obtained through simulation using PSIM, while Fig. 15 shows the signals generated by the emulation hardware. It is observed that the settling time is less than 400μs, matching the emulation results, which confirms the accuracy of the hardware in replicating the system dynamics.
  Additionally, it is observed that the signal delivered by the hardware remains within the same average values shown in the scaled data presented at the bottom of Fig. 14 (capacitor voltage → red waveform, inductor current → blue waveform). This consistency verifies the correct scaling and emulation of the converter, ensuring that the hardware faithfully reproduces the expected behavior of the system under these dynamic conditions.
  The system was evaluated using a 100 kHz switching signal to assess its performance at high operating frequencies. At this switching frequency, the converter exhibited the expected behavior in both the capacitor voltage and the inductor current, as illustrated in Fig. 16.
• **HIL SEPIC converter:** Additionally, the behavior of the SEPIC converter was emulated, which presents a higher level of complexity due to the presence of an additional inductor and capacitor. This converter can become unstable under certain operating conditions and improper component selection, making careful design critical. Furthermore, the SEPIC converter has a significantly higher gain factor compared to Boost and Buck-Boost converters, which implies higher scaling values. This results in output signals with lower emulation resolution due to the limitations of the digital conversion system.
  The validation was carried out using the following parameters: an input voltage of 12 V, a capacitor $C_{1}$ of 100μF, a capacitor $C_{2}$ of 300μF, an inductance $L_{1}$ of 330 μH, an inductance $L_{2}$ of 100 μH, and a load resistance of 1 Ω. For these values, the following scaling factors were determined: 52 mV/V for the voltage on $C_{1}$, 208 mV/V for the voltage on $C_{2}$, 13 mV/A for the current in $L_{1}$, and 43.4 mV/A for the current in $L_{2}$.
  Since the currents have the lowest scaling values, caused by the high current levels the converter can reach, and considering that the resolution of the DAC used is approximately 11.72 mV, it is not possible to observe the ripple in the inductor currents. However, the average values of these currents are visible, allowing the general behavior of the emulated converter to be validated. Therefore, for this simulation, the voltages on the two capacitors are presented.
  In Fig. 17, the simulation results are presented for a switching frequency of 20 kHz using the same parameters as in the emulation. In the upper section, both capacitors reach an average of 12 V. The lower section shows the two waveforms scaled according to their operating ranges; since the voltage across the output capacitor can reach higher values, its scaling factor is larger, resulting in a significantly lower average value in the scaled signal. Fig. 18 shows the corresponding emulation results, where the two signals represent the voltage across capacitor $C_{1}$ (red) and the voltage across capacitor $C_{2}$ (blue). For a duty cycle of 50%, these signals exhibit average values of approximately 2.7 V and 0.62 V, respectively.
  A change in the duty cycle of the converter from 50% to 60% is performed to validate the emulation. It is observed that the emulated signals (Fig. 20) adequately replicate the general dynamic behavior and the average values obtained in the simulations (Fig. 19). Both figures show a similar transient response, although the emulated signals exhibit smoother transitions and slightly damped oscillations. The average values of the voltages across the capacitors $C_{1}$ and $C_{2}$ are close between both graphs, validating the hardware’s fidelity in replicating the system’s steady-state behavior. Additionally, the scaled signals confirm the proper adjustment to the DAC’s operating range, although the emulated signals have a lower resolution.
  For the SEPIC converter operating at a 50% duty cycle, the simulation shows that the voltage across capacitor C1 stabilizes at approximately 12.0 V, while C2 reaches around 11.8 V. The emulated analog signals, scaled to the DAC output range, yield corresponding voltages of approximately 0.7 V for C1 and 2.5 V for C2, reflecting the expected ratios given the scaling factors used (52 mV/V for C1 and 211 mV/V for C2). The voltage ripple observed in the simulation is approximately ±0.3 V for C1 and ±0.2 V for C2. In the emulated signals, ripple is less pronounced due to the combined effects of DAC resolution (8-bit → 11.7 mV/step) and signal smoothing. The effective ripple measured in the emulation was approximately ±0.06 V (C1) and ±0.03 V (C2).
  After increasing the duty cycle to 60%, the capacitor voltage C1 rises to 18 V while the capacitor voltage C2 remains at 12 V. The emulated signals increase to approximately 0.93 V (C1 blue line) and 2.5 V (C2 green line), maintaining the scaling proportions. The transient response and settling times remain consistent between simulation and emulation, with the emulated waveforms exhibiting slightly smoother transitions due to signal quantization and hardware filtering.
  Additionally, tests were conducted on the SEPIC converter operating at a switching frequency of 100 kHz, as shown in Fig. 21. Under these conditions, a considerable reduction in the steady-state ripple is observed, along with a dynamic response that matches the behavior predicted by simulation. However, the results also reveal the appearance of subharmonic components in the transient response. This phenomenon is attributed to the absence of input oversampling and to the increased sampling delay caused by the higher computational load of the model at this switching frequency. Without oversampling, the input sampling rate approaches the converter’s switching period, which can cause aliasing effects that distort the accurate reconstruction of the duty cycle. These distortions are more noticeable during rapid transients, where the combined effect of model complexity and limited sampling resolution amplifies subharmonic oscillations.
  In the current implementation, the PWM input signal is not measured while the model equations are being executed, but rather in the time interval between consecutive executions of the HIL model. During this interval, multiple instantaneous PWM readings are taken with a temporal resolution of approximately 10 ns, and these samples are processed using the IOM. The IOM integrates and corrects variations detected in the duty cycle within that period, producing a representative value of the control signal state. This processed value is then used to execute the next model step, whose output is subsequently sent to the DAC before repeating the complete cycle.
  Although this approach improves duty-cycle estimation compared to taking a single reading per simulation step, all PWM acquisition and processing occur sequentially with respect to model execution, without any asynchronous or parallel acquisition mechanism. As a result, while rapid changes within the sampling interval can be detected thanks to the high temporal resolution of the readings, the system’s responsiveness remains limited by the total duration of the model step and the accumulated delay between captures and updates. Under high switching frequencies or computationally demanding models, this limitation can cause aliasing, errors in duty-cycle reconstruction, and the appearance of sub-harmonic components in the dynamic response.
  This restriction is mainly associated with the firmware architecture. A possible improvement would be to decouple PWM acquisition from the simulation loop by using hardware capture or edge-triggered interrupts to log the rising and falling times of the signal, thus enabling a truly asynchronous and higher-fidelity duty-cycle reconstruction. Additionally, reducing the model computation time or enabling overclock on the Teensy 4.1 could provide extra timing margin, although the latter may require active cooling to ensure thermal stability.

## Conclusions

This work demonstrated the feasibility of implementing a low-cost real-time HIL emulator for DC–DC converters using the Teensy 4.1 microcontroller. The proposed platform successfully reproduced the steady-state and dynamic behavior of Buck and SEPIC converters, showing a high degree of correlation with PSIM simulation results. The integration of the IOM allowed improved duty-cycle estimation from high-frequency PWM control signals, even though sampling and processing were performed sequentially between model steps.

The system achieved a model step time of 1μs, enabling operation with switching frequencies up to 100 kHz and providing at least 10 integration steps per switching period, in accordance with commonly accepted fidelity criteria. The use of simple 8-bit R–2R ladder DACs proved sufficient for representing scaled analog variables, with output ripple and average values consistent with simulation predictions.

However, the absence of fully asynchronous PWM acquisition limited the ability to capture rapid transitions and introduced aliasing and sub-harmonic oscillations under high-frequency or computationally demanding scenarios. These effects were especially noticeable at 100 kHz switching frequency, where the increased model complexity and processing delay reduced effective temporal resolution.

Future work will focus on decoupling PWM acquisition from model execution through hardware capture modules or interrupt-based edge detection, enabling true oversampling with sub-microsecond resolution. Additional improvements may include optimization of numerical algorithms, exploration of higher DAC resolution, and the use of overclocked operation to reduce step latency (potentially requiring active cooling). The proposed approach offers a versatile, compact, and cost-effective HIL platform suitable for rapid prototyping and validation of power converter control strategies.

## CRediT authorship contribution statement

**Alejandro Gaviria-Cano:** Validation, Software. **Cristian Escudero-Quintero:** Writing – review & editing, Resources. **Jose David López-Suárez:** Validation. **Juan Pablo Villegas-Ceballos:** Software, Methodology, Conceptualization. **Elkin Edilberto Henao-Bravo:** Validation, Conceptualization. **Sergio Ignacio Serna-Garcés:** Formal analysis, Conceptualization.

## Declaration of competing interest

The authors declare that they have no known competing financial interests or personal relationships that could have appeared to influence the work reported in this paper.
